# Inhibitory Effect of Thymol on Tympanostomy Tube Biofilms of Methicillin-Resistant *Staphylococcus aureus* and Ciprofloxacin-Resistant *Pseudomonas aeruginosa*

**DOI:** 10.3390/microorganisms10091867

**Published:** 2022-09-19

**Authors:** Eu-Ri Jo, Jeonghyun Oh, Sung Il Cho

**Affiliations:** 1 Department of Otolaryngology-Head and Neck Surgery, College of Medicine, Chosun University, Gwangju 61453, Korea; 2 Department of Biomedical Sciences, Graduate School, Chosun University, Gwangju 61452, Korea

**Keywords:** thymol, methicillin-resistant *Staphylococcus aureus*, ciprofloxacin-resistant *Pseudomonas aeruginosa*, biofilm, tympanostomy tube

## Abstract

The formation of antibiotic-resistant strain biofilms in tympanostomy tubes results in persistent and refractory otorrhea. In the present study, we investigated the in vitro antibiofilm activity of thymol against biofilms formed by methicillin-resistant *Staphylococcus aureus* (MRSA) and ciprofloxacin-resistant *Pseudomonas aeruginosa* (CRPA), using live and dead bacterial staining and adhesion, biofilm formation, biofilm eradication, and biofilm hydrolytic activity assays. The antibiofilm activity of thymol against tympanostomy tube biofilms formed by MRSA and CRPA strains was examined using a scanning electron microscope. In response to thymol treatment, we detected significant concentration-dependent reductions in the viability and adhesion of MRSA and CRPA. Exposure to thymol also inhibited the formation of both MRSA and CRPA biofilms. Furthermore, thymol was observed to enhance the eradication of preformed mature biofilms produced by MRSA and CRPA and also promoted a reduction in the rates of MRSA and CRPA hydrolysis. Exposure to thymol eradicated extracellular polysaccharide present in the biofilm matrix produced by MRSA and CRPA. Additionally, thymol was observed to significantly eradicate MRSA and CRPA biofilms that had formed on the surface on tympanostomy tubes. Collectively, our findings indicate that thymol is an effective inhibitor of MRSA and CRPA biofilms, and accordingly has potential utility as a therapeutic agent for the treatment of biofilm-associated refractory post-tympanostomy tube otorrhea resulting from MRSA and CRPA infection.

## 1. Introduction

Tympanostomy tubes are small tubes that are inserted into the tympanic membrane in order to prevent the accumulation of effusion in the middle ear and to maintain aeration in the middle ear, the placement of which is commonly performed for the treatment of otitis media with effusion [1]. However, the insertion of these devices often leads to ear infections, among which otorrhea is the most common complication in children [2]. Otorrhea that continues for periods exceeding 8 weeks is classified as persistent otorrhea, the incidence of which is 3.8% in those who have undergone operations [3]. Among the bacteria associated with this condition, *Staphylococcus aureus*, *Pseudomonas aeruginosa*, and *Haemophilus influenzae* are the most common isolated pathogens in post-tympanostomy tube otorrhea [4,5], with the biofilm formation of these species resulting in persistent and refractory otorrhea [6]. In particular, the increased prevalence of antibiotic-resistant strains in otitis media such as methicillin-resistant *S. aureus* (MRSA) and ciprofloxacin-resistant *P. aeruginosa* (CRPA), has led to an increase in biofilm formation, resulting in refractory post-tympanostomy tube otorrhea [7,8,9]. Although topical application of antibiotics has been used in an effort to inhibit biofilm development on tympanostomy tubes, this therapeutic approach has generally been found to be ineffective, particularly if those biofilms are produced by antibiotic-resistant strains [10].

Tea-tree oil and other essential oils have been reported to possess antimicrobial activity against *S. aureus*, *P. aeruginosa*, and MRSA biofilms [11,12,13]. However, clinical application of the agents is not yet available.

Thymol (2-isopropyl-5-methylphenol) is a monoterpene phenol extracted from plants in the families Lamiaceae, Verbenaceae, and Scrophulariaceae [14], and it has been reported to have a broad spectrum of beneficial therapeutic properties, including antioxidant, anticancer, anti-inflammatory, antibacterial, antifungal, antitubercular, and antiparasitic activities [15,16,17]. Thymol has also been established to have antimicrobial and anti-inflammatory activities against multi-drug resistant microorganisms [14,18], The strong antibacterial properties of thymol have been reported in the treatment of respiratory infections, oral cavity infections, and intestinal diseases [19,20]. 

Thymol is accordingly considered to have potential therapeutic application in the treatment of MRSA infections. In the present study, we sought to assess the antibiofilm activity of thymol on biofilms produced by MRSA and CRPA strains and thereby establish its potential utility in the treatment of refractory post-tympanostomy tube otorrhea. 

## 2. Materials and Methods

### 2.1. Bacterial Strains and Drug

Each MRSA and CRPA strains used in this study were obtained from a different patient with post-tympanostomy tube otorrhea at Chosun University Hospital. Clinical isolates were subcultured for 24 h in blood agar plates (Hanil-KOMED, Seongnam, Korea). They were inoculated into the GP and GN cards of VITEK 2 system (bioMérieux Inc., Durham, NC, USA), and then tested for the identification of *S. aureus* and *P. aeruginosa* according to the recommendations of the manufacturer. The MRSA and CRPA strains were phenotypically characterized by antibiotic susceptibility test cards (AST-P601 for MRSA, AST-N225 for CRPA) of VITEK 2 system (bioMérieux Inc.). The MRSA and CRPA isolates were cultured at 37 °C on Mueller–Hinton agar (Difco Laboratories, Sparks, MD, USA) plates. Thymol was purchased from ThermoFisher Scientific (Waltham, MA, USA), a stock solution (250 mg/mL) of which was prepared by dilution in ethanol (1.25%, Merck KGaA, Darmstadt, Germany).

### 2.2. Determination of the Minimum Inhibitory Concentration of Thymol

MRSA and CRPA bacterial suspensions at concentrations of approximately 10^8^ CFU/mL were prepared from fresh log-phase bacterial cultures, aliquots of which were mixed with different concentrations of thymol (0–6.25 mg/mL; serially diluted in Mueller–Hinton broth) in the wells of sterile 96-well plates. The plates were incubated at 37 °C for 24 h, after which the series of dilution wells were observed for microbial growth. The minimum inhibitory concentration (MIC) was defined as the lowest concentration of thymol at which there was no visible growth of bacteria (i.e., an absence of visible turbidity) after 20 to 24 h of growth, and the difference in measured OD_600_ was less than 0.01. 

### 2.3. Live and Dead Bacterial Cell Staining

Culture suspensions of either MRSA or CRPA (5 mL, 10^7^ CFU/mL) were incubated in culture tubes with different concentrations of thymol (0, 0.1, 0.3, 0.5, 1, and 2 mg/mL for MRSA; 0, 0.3, 0.5, 1, 2, and 3 mg/mL for CRPA) in Mueller–Hinton broth at 37 °C for 24 h with shaking at 200 rpm. Bacterial cell staining was performed using a LIVE/DEAD *Bac*Light bacterial viability kit (Invitrogen, Carlsbad, CA, USA). Aliquots (1 mL) of MRSA or CRPA bacterial suspensions cultured with thymol were transferred to microtubes and centrifuged at 10,000 rpm for 10 min. The supernatants thus obtained were discarded and the remaining pellets were resuspended in 1 mL of phosphate-buffered saline (PBS). The resuspension samples were again centrifuged, supernatants were discarded, and pellets were resuspended in 200 µL of PBS. Equal volumes of compounds A and B (provided with the kit) were combined, and 3 µL of this dye mixture was added to 1 mL of the bacterial suspension. Samples were incubated at room temperature for 15 min in the dark, after which, 5 µL of the stained bacterial suspensions were placed on a slide and mounted with mounting medium (Biomeda Corp., San Jose, CA, USA). The specimens were observed using a confocal microscope (Carl Zeiss, Oberkochen, Germany), and the images obtained were analyzed using Zeiss microscope image software ZEN (Carl Zeiss).

### 2.4. Adhesion Assay

Culture suspensions of MRSA or CRPA (2 mL, 10^7^ CFU/mL) and different concentrations of thymol (0, 0.1, 0.3, 0.5, 1, and 2 mg/mL for MRSA; 0, 0.3, 0.5, 1, 2, and 3 mg/mL for CRPA) in Mueller–Hinton broth were added to the wells of six-well plates and incubated for 30 min at 37 °C. The well contents were then washed three times with PBS to remove unbound bacteria, and the adherent bacteria were fixed by heating at 60 °C for 30 min, followed by staining with 2 mL of 1% crystal violet solution (Sigma, Saint Louis, MO, USA) for 5 min. Thereafter, the plates were washed three times with PBS and imaged using an ECLIPSE Ti2-E microscope (Nikon, Tokyo, Japan). To determine the mean number of adherent cells, we counted cells in at least five fields of view in each well at ×200 magnification.

### 2.5. Biofilm Formation Assay

Culture suspensions of MRSA or CRPA (200 µL, 10^7^ CFU/mL) and different concentrations of thymol (0, 0.1, 0.3, 0.5, 1, and 2 mg/mL for MRSA; 0, 0.3, 0.5, 1, 2, and 3 mg/mL for CRPA) in tryptic soy broth (TSB) containing 1% glucose were incubated in 96-well plates for 24 h at 37 °C. The plates were then washed three times with PBS and fixed at 60 °C for 30 min. The biomass of the remaining biofilm was stained with 200 µL of 1% crystal violet solution (Sigma) for 15 min, followed by three washes with PBS, and then air dried at room temperature. Having dried, 200 µL of 95% ethanol was added to each well and the absorbance of the well contents was measured at 570 nm using a BioTek plate reader (BioTek, Winooski, VT, USA).

### 2.6. Determination of the Minimum Biofilm Eradication Concentration of Thymol 

A biofilm eradication assay was performed using the minimum biofilm eradication concentration (MBEC) assay Biofilm Inoculator with a 96-well base (Innovotech Inc., Edmonton, AB, Canada). Culture suspensions of MRSA or CRPA (180 µL, 10^7^ CFU/mL) in TSB containing 1% glucose were inoculated into a Calgary device (Innovotech Inc.), comprising a 96-well plate with a lid containing pegs for biofilm establishment, and incubated for 24 h at 37 °C. Thereafter, the lid of the Calgary device was removed, washed twice with PBS, and transferred to another 96-well plate containing 200 µL of different concentrations of thymol (0, 0.1, 0.3, 0.5, 1, and 2 mg/mL for MRSA; 0, 0.3, 0.5, 1, 2, and 3 mg/mL for CRPA) and incubated for 24 h at 37 °C. The lid was then transferred to a further fresh 96-well plate containing 200 µL of fresh Mueller–Hinton broth and incubated for 24 h at 37 °C. The MBEC was defined as the minimum concentration of antimicrobial that eradicates the biofilm, with values being determined based on wells showing an absence of turbidity, and optical density measurements being obtained at 600 nm using a plate reader.

For viable cell counts, pegs were rinsed twice with PBS and placed into fresh plate containing PBS. The plates with a lid containing pegs were sonicated in a Ultrasonics 5510 sonic water bath (Branson, Danbury, CT, USA) for 30 min. Serial dilutions were performed by plating on LB agar plates. After incubation for 24 h at 37 °C, colony-forming units per milliliter (CFU/mL) was determined by counting the viable colony.

### 2.7. Biofilm Hydrolysis Assay

Biofilm hydrolysis was assessed using fluorescein diacetate (FDA; Sigma, Saint Louis, MO, USA), a stock solution (2 mg/mL) of which was prepared by dissolving in acetone. For FDA determinations of hydrolytic activity against biofilms, 200 µL aliquots of MRSA or CRPA culture suspensions (10^7^ CFU/mL) in TSB containing 1% glucose were inoculated and incubated for 24 h at 37 °C in 96-well plates. The contents of plate wells were then decanted, and the wells were washed two times with PBS, followed by the addition of 200 µL of different concentrations of thymol (0, 0.1, 0.3, 0.5, 1, and 2 mg/mL for MRSA; 0, 0.3, 0.5, 1, 2, and 3 mg/mL for CRPA), and subsequent incubation at 37 °C for 24 h. Thereafter, the plates were washed three times with PBS followed by the addition of 200 µL of FDA (final concentration, 10 µg/mL) in PBS and incubation at 37 °C for 3 h. The amount of fluorescein released (due’to hydrolytic activity of extracellular enzymes) was measured at 490 nm using a plate reader. 

### 2.8. Scanning Electron Microscope Analysis of Bacterial Biofilms

Tympanostomy tubes (Paparella type; Medtronic, Minneapolis, MN, USA) were placed in the wells of 96-well plates containing 1% glucose-supplemented TSB and previously prepared MRSA and CRPA suspensions (200 μL, 10^7^ CFU/mL). Following incubation at 37 °C for 24 h, the contents of plate wells were decanted and the tympanostomy tubes were washed two times with PBS to eliminate unbound bacteria. Thereafter solutions of different concentrations of thymol (200 μL; 0, 0.1, 0.3, 0.5, 1, and 2 mg/mL for MRSA; 0, 0.3, 0.5, 1, 2, and 3 mg/mL for CRPA) were added to wells followed by incubation at 37 °C for 24 h. Thereafter, the tympanostomy tubes were transferred to the wells of 12-well plates, in which they were fixed with 2.5% glutaraldehyde (Daejung, Siheung, Korea) for 30 min and then washed twice with PBS. The plates containing tympanostomy tubes were immersed in an ethanol series (50%, 60%, 70%, 80%, 90%, and 100%, 10 min at each concentration), air dried, coated using an E-1030 ion sputtering coating device (Hitachi High-Technologies Corp., Tokyo, Japan), and observed under an S-4800 Field Emission Scanning Electron Microscope (Hitachi High-Technologies Corp., Tokyo, Japan) for 2 h.

### 2.9. Fluorescence Microscopic Observation and Quantification of Biofilm Matrix

Observation and quantification of MRSA and CRPA biofilm matrix were investigated with wheat germ agglutinin (WGA)-Alexa Fluor 488 conjugate (Invitrogen, Carlsbad, CA, USA) which binds specifically to polysaccharide adhesion (poly-N-acetyl glucosamine) in biofilm matrix formed by bacteria. Culture suspensions of MRSA or CRPA (2 mL, 10^7^ CFU/mL) were seeded onto sterile 12 mm glass coverslip in 12 wells plates at 37 °C for 24 h, and preformed MRSA and CRPA biofilms were treated with solutions of different concentrations of thymol (0, 0.1, 0.3, 0.5, 1, and 2 mg/mL for MRSA; 0, 0.3, 0.5, 1, 2, and 3 mg/mL for CRPA) in TSB containing 1% glucose at 37 °C for 24 h. The wells were then washed twice with PBS, and stained with 2 mL of 5 µg/mL WGA Alexa 488 in PBS at 37 °C for 20 min in the dark. Thereafter, the wells were washed twice with PBS and fixed with 4% formaldehyde at 37 °C for 15 min. The fixed coverslip was placed on slides using Fluorescent Mounting Medium with DAPI (GBI Labs, Bothell, WA, USA). Immunofluorescence was detected by confocal microscopy (Carl Zeiss, Oberkochen, Germany), and analyzed using Zeiss microscope image software ZEN (Carl Zeiss).

For quantification of the biofilm matrix stained with WGA Alexa 488, culture suspensions of MRSA or CRPA (180 µL, 10^7^ CFU/mL) in TSB containing 1% glucose were inoculated in a 96-well black plate at 37 °C for 24 h. Preformed MRSA and CRPA biofilms were treated with solutions of different concentrations of thymol at 37 °C for 24 h, and were washed twice with PBS. Biofilm in each well was stained with 125 µL of 5 µg/mL WGA Alexa 488 in PBS at 37 °C for 20 min in the dark. Wells were washed twice with PBS and air dried at room temperature for 15 min. 125 µL of 33% acetic acid was added to each well. Thereafter, the 96-well plate was sonicated in a sonicator (Branson, Danbury, CT, USA) for 20 min. The WGA fluorescence was measured at the excitation wavelength of 495 nm and the emission wavelength of 520 nm by spectrofluorometer (Molecular Devices, San Jose, CA, USA).

### 2.10. Statistical Analysis

Results were analyzed using SPSS 25.0 software (SPSS Inc., Chicago, IL, USA). One-way ANOVA was used to analyze the data. Each experiment was performed five independent times. A *p*-value < 0.05 was considered indicative of statistical significance.

## 3. Results

### 3.1. Inhibitory Effect of Thymol against MRSA and CRPA

**Figure 1:**
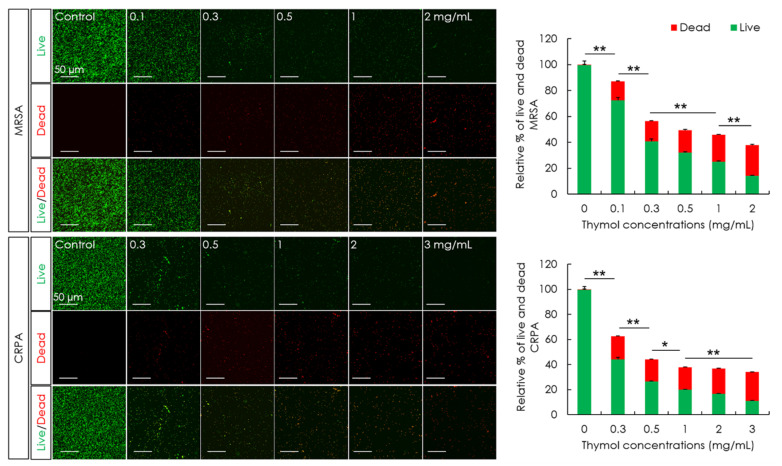
The effects of thymol on the viability of methicillin-resistant *Staphylococcus aureus* (MRSA) and ciprofloxacin-resistant *Pseudomonas aeruginosa* (CRPA). MRSA and CRPA viabilities were examined following 24 h exposure to different concentrations of thymol. Thymol promoted significant concentration-dependent reductions in live MRSA and CRPA cells (green), and increases in dead MRSA and CRPA cells (red). Data are shown as the means ± standard errors of the mean of five independent experiments (** *p* < 0.01; * *p* < 0.05).

The minimum inhibitory concentrations (MICs) of thymol against MRSA and CRPA strains following 24 h exposure to thymol were 0.40 and 1.56 mg/mL, respectively. The viability of MRSA and CRPA was examined based on live/dead bacterial staining and the viability at each concentration of thymol was calculated as the percentage relative to the control. Thymol accordingly revealed a significant concentration-dependent reduction in the green fluorescence of live MRSA and CRPA and a significant increase in the red fluorescence of dead cells following thymol treatment (*p* < 0.05, Figure 1).

### 3.2. Inhibitory Effect of Thymol against the Adhesion and Biofilm Formation of MRSA and CRPA

**Figure 2:**
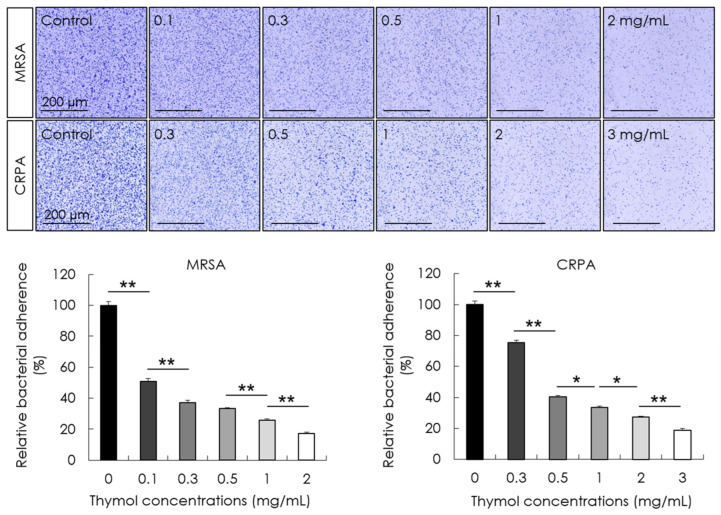
The effects of thymol on the adhesion of methicillin-resistant *Staphylococcus aureus* (MRSA) and ciprofloxacin-resistant *Pseudomonas aeruginosa* (CRPA). The adherent MRSA and CRPA strains were stained using crystal violet following a 30 min treatment with thymol (top). Thymol significantly reduced the adhesion of MRSA and CRPA in a concentration-dependent manner. Data are shown as the means ± standard errors of the mean of five independent experiments (** *p* < 0.01; * *p* < 0.05).

**Figure 3:**
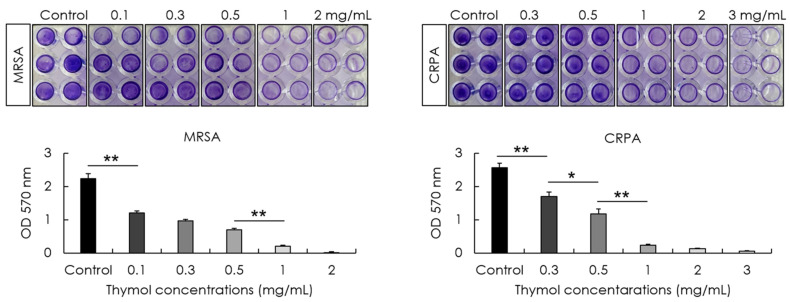
The activity of thymol against the formation of biofilms produced by methicillin-resistant *Staphylococcus aureus* (MRSA) and ciprofloxacin-resistant *Pseudomonas aeruginosa* (CRPA). Biofilm formation was examined following 24 h exposure to thymol. Thymol significantly inhibited the rates of MRSA and CRPA biofilm formation in a concentration-dependent manner. Data are shown as the means ± standard errors of the mean of five independent experiments (** *p* < 0.01, * *p* < 0.05).

The effects of different concentrations of thymol on bacterial adhesion and biofilm formation were determined following 30 min and 24 h exposures, respectively. The bacterial adhesion at each concentration of thymol was calculated as the percentage relative to the control. Compared with the control treatment, we found that thymol promoted a significant concentration-dependent reduction in the adhesion of MRSA and CRPA (*p* < 0.05, Figure 2) and significant concentration-dependent inhibition of the rate of biofilm formation by these strains (*p* < 0.05, Figure 3).

### 3.3. Eradication Effect of Thymol against MRSA and CRPA Biofilms

**Figure 4:**
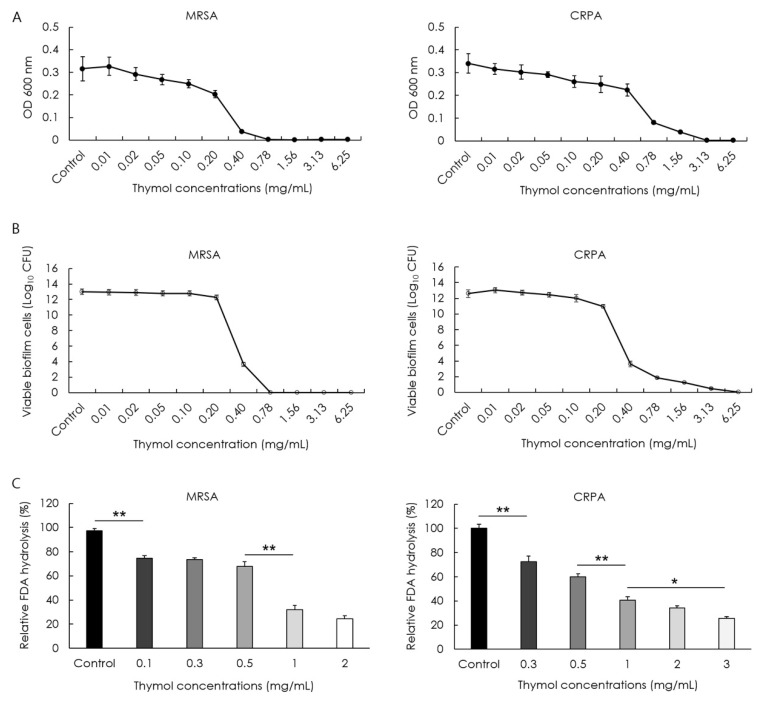
The activity of thymol against the preformed biofilms of methicillin-resistant *Staphylococcus aureus* (MRSA) and ciprofloxacin-resistant *Pseudomonas aeruginosa* (CRPA). Eradication and hydrolytic activity were determined following treatment with thymol for 24 h. The rates of MRSA and CRPA biofilm eradication increased in response to exposure to increasing concentrations of thymol. The minimum biofilm eradication concentrations (MBECs) of thymol by optical density measurements against preformed MRSA and CRPA biofilms were 0.78 and 3.13 mg/mL, respectively (**A**). The MBEC of thymol by viable cell counts against preformed MRSA and CRPA biofilms were 0.78 and 6.25 mg/mL, respectively (**B**). The hydrolytic activity of biofilms produced by MRSA and CRPA decreased significantly in response to exposure to increasing concentrations of thymol (**C**). Data are shown as the means ± standard errors of the mean of five independent experiments (CFU; colony-forming units, FDA; fluorescein diacetate, ** *p* < 0.01; * *p* < 0.05).

**Figure 5:**
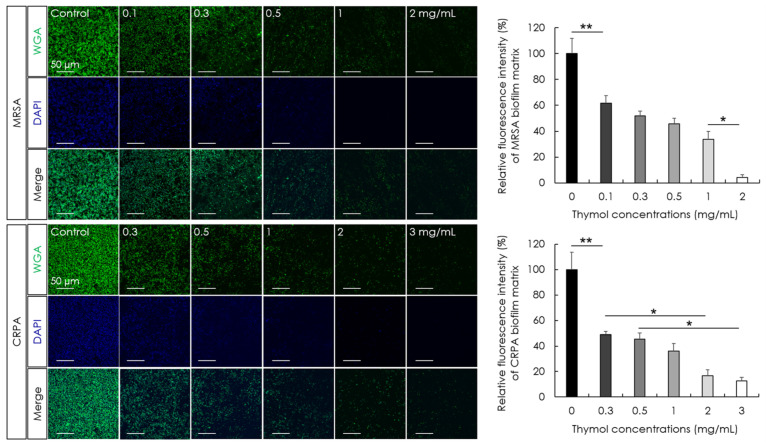
The activity of thymol against the extracellular matrix of biofilms produced by methicillin-resistant *Staphylococcus aureus* (MRSA) and ciprofloxacin-resistant *Pseudomonas aeruginosa* (CRPA). The extracellular matrix was determined by staining with WGA-Alexa 488 following treatment with thymol for 24 h. The extracellular matrix of biofilms produced by MRSA and CRPA decreased significantly in response to exposure to increasing concentrations of thymol. Data are shown as the means ± standard errors of the mean of five independent experiments (WGA; wheat germ agglutinin, DAPI; 4′,6-diamidino-2-phenylindole, ** *p* < 0.01; * *p* < 0.05).

The minimum biofilm eradication concentrations (MBECs) of thymol and effects on the hydrolytic activity and extracellular matrix of MRSA and CRPA biofilms were determined following 24 h exposure. We accordingly obtained thymol MBEC values of 0.78 and 3.13 mg/mL by optical density measurements against the preformed mature biofilms produced by MRSA and CRPA strains, respectively (Figure 4A). To confirm the eradication effect of thymol based on optical density measurements, viable cell counts were determined after 24 h of growth. The MBEC values of thymol were 0.78 and 6.25 mg/mL by colony-forming unit (CFU) measurements against the preformed mature biofilms produced by MRSA and CRPA strains, respectively (Figure 4B). The enzymatic activity of microbial populations and overall microbial activity were assessed based on fluorescein diacetate (FDA) release assays. Products of FDA hydrolysis correlate with the microbial activity of MRSA and CRPA. We found that exposure to thymol promoted a significant concentration-dependent reduction in the rates of MRSA and CRPA biofilms hydrolysis (*p* < 0.05, Figure 4C). The polysaccharide amount was investigated using a WGA conjugate that targets the poly-N-acetyl glucosamine fraction of the biofilm matrix. Exposure to thymol promoted a significant concentration-dependent reduction in the biofilm matrix of MRSA and CRPA (*p* < 0.05, Figure 5). These results indicated that thymol eradicated the preformed biofilms produced by MRSA and CRPA strains.

### 3.4. Biofilm-Destruction Activity of Thymol against Biofilm-Coated Tympanostomy Tubes

**Figure 6:**
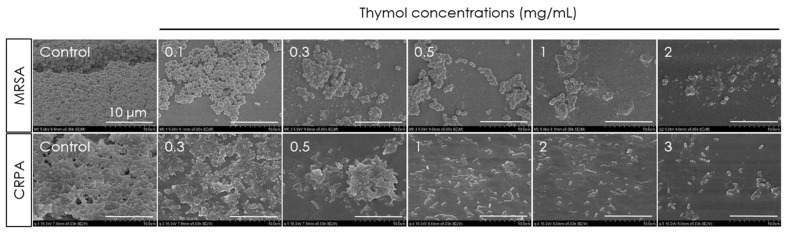
Scanning electron micrographs of methicillin-resistant *Staphylococcus aureus* (MRSA) and ciprofloxacin-resistant *Pseudomonas aeruginosa* (CRPA) biofilms on the surface of tympanostomy tubes after 24 h exposure to thymol. Thymol was found to concentration-dependently reduce the MRSA and CRPA biofilms and colonies.

The antibiofilm activity of thymol against the development of MRSA and CRPA biofilms on tympanostomy tube surfaces was compared based on scanning electron microscopy observations following 24 h treatments with different concentrations of thymol. Examination of the surfaces of control group tympanostomy tubes revealed the occurrence of numerous MRSA and CRPA colonies and biofilm formation, whereas we observed marked concentration-dependent reductions in the numbers of MRSA and CRPA colonies and biofilm development in the thymol-treated groups (Figure 6).

## 4. Discussion

Prolonged infection and chronic otorrhea subsequent to tympanostomy tube placement are frequently associated with biofilm-mediated infection [21], with strains of *S. aureus* and *P. aeruginosa* often identified as the causal bacteria [22]. Biofilms comprise microbial communities in which bacteria co-exist within an extracellular polysaccharide matrix, and differences in the microstructure of these biofilms contribute to determining their susceptibility/resistance to different antibiotics [23,24]. The biofilms produced by antibiotic-resistant strains, such as those of MRSA and CRPA, are notably more resistant, thereby presenting new therapeutic challenges [8,9].

The plant-derived monoterpene phenol thymol shows bacteriostatic activity against both Gram-positive and -negative bacteria [25], with antibacterial effects attributable to the disruption of the bacterial plasma membrane and subsequent leakage of intracellular contents [26]. Moreover, thymol has been demonstrated to have inhibitory effects against biofilms produced by *Chromobacterium violaceum,* attributable to the inhibition of quorum sensing [27], and shows similar inhibitory activity against fungal biofilm produced by *Candida albicans* [28].

In the present study, we found that thymol has inhibitory activity at different steps in the development of biofilms produced by strains of MRSA and CRPA. Thymol had antimicrobial activity against planktonic and biofilm bacterial cells. In addition, thymol proved to be effective in reducing the adhesion, biofilm formation, hydrolytic activity, and extracellular matrix of MRSA and CRPA biofilms and also eradicated preformed mature biofilms on the surface of tympanostomy tubes. The mechanism underlying the antibiofilm effect of thymol can be interference with the surface adherence of antibiotic-resistant strains and the destruction of the cell membrane structure [29]. Thymol leads to the destabilization of the phospholipid bilayer and results in the destruction of the microbial membrane [12]. In addition, thymol can attenuate the virulence of MRSA by *sarA* inhibition and inhibit extracellular polysaccharide present in the biofilm matrix [30]. Thymol alters cellular communication pathways such as quorum sensing and inhibits the production of poly-N-acetyl glucosamine (PNAG) in MRSA biofilm matrix [31]. Thymol also inhibits an adhesin CdrA which contributes to CRPA biofilm formation and stabilization [32].

Our results suggest that coating tympanostomy tubes with thymol can effectively prevent the initial adherence of MRSA and CRPA biofilms to the tubes. Topical application of antimicrobial eardrops is a standard treatment for post-tympanostomy tube otorrhea, as this enables the delivery of a considerably higher concentrations to the ear [33]. From the perspective of practical eardrop application, we found that 0.78 and 6.25 mg/mL thymol would be optimal concentrations for the prevention of biofilm formation and the eradication of preformed MRSA and CRPA biofilms on tympanostomy tubes, respectively.

Given that topically applied eardrops can pass through tympanostomy tubes, reach the middle ear, and potentially result in damage of the inner ear, the safety of these products is an important issue. However, in both animal and human trials, thymol has shown no evidence of deleterious effects, and is accordingly considered to be safe [34]. Indeed, thymol is classified as “Generally Recognized As Safe” by the United States Food and Drug Administration when used in foods for human consumption, or as food additives [35]. We used an alcoholic solution to prepare a stock solution of thymol. Therefore, further studies on the formulaton strategy of thymol are required to assess whether it is applicable to the middle ear. In summary, in the present study, we investigated the in vitro antibiofilm activity of thymol against the biofilms produced by MRSA and CRPA, and demonstrated that thymol effectively prevents biofilm formation and eradicates preformed biofilms. Thymol simultaneously caused both cell lysis and biofilm matrix disruption. Our findings indicate that thymol has considerable potential as a therapeutic agent for the treatment of refractory post-tympanstomy otorrhea attributable to MRSA and CRPA biofilm infections. However, further clinical studies are necessary to verify this conclusion.

## Data Availability

Data are available upon request.

## References

[1] Rosenfeld R.M., Shin J.J., Schwartz S.R., Coggins R., Gagnon L., Hackell J.M., Hoelting D., Hunter L.L., Kummer A.W., Payne S.C. (2016). Clinical practice guideline: Otitis media with effusion (update). Otolaryngol. Head Neck Surg..

[2] Steele D.W., Adam G.P., Di M., Halladay C.W., Balk E.M., Trikalinos T.A. (2017). Prevention and treatment of tympanostomy tube otorrhea: A meta-analysis. Pediatrics.

[3] van Dongen T.M.A., Damoiseaux R.A.M.J., Schilder A.G.M. (2018). Tympanostomy tube otorrhea in children: Prevention and treatment. Curr. Opin. Otolaryngol. Head Neck Surg..

[4] Mandel E.M., Casselbrant M.L., Kurs-Lasky M. (1994). Acute otorrhea: Bacteriology of a common complication of tympanostomy tubes. Ann. Otol. Rhinol. Laryngol..

[5] van Dongen T.M., Venekamp R.P., Wensing A.M., Bogaert D., Sanders E.A., Schilder A.G. (2015). Acute otorrhea in children with tympanostomy tubes: Prevalence of bacteria and viruses in the post-pneumococcal conjugate vaccine era. Pediatr. Infect. Dis. J..

[6] Karlan M.S., Skobel B., Grizzard M., Cassisi N.J., Singleton G.T., Buscemi P., Goldberg E.P. (1980). Myringotomy tube materials: Bacterial adhesion and infection. Otolaryngol. Head Neck Surg..

[7] Kim S.H., Kim M.G., Kim S.S., Cha S.H., Yeo S.G. (2015). Change in detection rate of methicillin-resistant *Staphylococcus aureus* and *Pseudomonas aeruginosa* and their antibiotic sensitivities in patients with chronic suppurative otitis media. J. Int. Adv. Otol..

[8] Jotić A., Božić D.D., Milovanović J., Pavlović B., Ješić S., Pelemiš M., Novaković M., Ćirković I. (2016). Biofilm formation on tympanostomy tubes depends on methicillin-resistant *Staphylococcus aureus* genetic lineage. Eur. Arch. Otorhinolaryngol..

[9] Jang C.H., Cho Y.B., Choi C.H. (2012). Effect of ion-bombarded silicone tympanostomy tube on ciprofloxacin-resistant *Pseudomonas aeruginosa* biofilm formation. Int. J. Pediatr. Otorhinolaryngol..

[10] Ciofu O., Rojo-Molinero E., Macià M.D., Oliver A. (2017). Antibiotic treatment of biofilm infections. APMIS.

[11] Park H., Jang C.H., Cho Y.B., Choi C.H. (2007). Antibacterial effect of tea-tree oil on methicillin-resistant *Staphylococcus aureus* biofilm formation of the tympanostomy tube: An in vitro study. In Vivo.

[12] Gómez-Sequeda N., Cáceres M., Stashenko E.E., Hidalgo W., Ortiz C. (2020). Antimicrobial and antibiofilm activities of essential oils against *Escherichia coli* O157:H7 and methicillin-resistant *Staphylococcus aureus* (MRSA). Antibiotics.

[13] Kavanaugh N.L., Ribbeck K. (2012). Selected antimicrobial essential oils eradicate Pseudomonas spp. and *Staphylococcus aureus* biofilms. Appl. Environ. Microbiol..

[14] Yuan Z., Dai Y., Ouyang P., Rehman T., Hussain S., Zhang T., Yin Z., Fu H., Lin J., He C. (2020). Thymol inhibits biofilm formation, eliminates pre-existing biofilms, and enhances clearance of methicillin-resistant *Staphylococcus aureus* (MRSA) in a mouse peritoneal implant infection model. Microorganisms.

[15] Xu J., Zhou F., Ji B.P., Pei R.S., Xu N. (2008). The antibacterial mechanism of carvacrol and thymol against *Escherichia coli*. Lett. Appl. Microbiol..

[16] de Castro R.D., de Souza T.M., Bezerra L.M., Ferreira G.L., Costa E.M., Cavalcanti A.L. (2015). Antifungal activity and mode of action of thymol and its synergism with nystatin against Candida species involved with infections in the oral cavity: An in vitro study. BMC Complement. Altern. Med..

[17] Sahoo C.R., Paidesetty S.K., Padhy R.N. (2021). The recent development of thymol derivative as a promising pharmacological scaffold. Drug Dev. Res..

[18] Marchese A., Orhan I.E., Daglia M., Barbieri R., Di Lorenzo A., Nabavi S.F., Gortzi O., Izadi M., Nabavi S.M. (2016). Antibacterial and antifungal activities of thymol: A brief review of the literature. Food Chem..

[19] Salehi B., Mishra A.P., Shukla I., Sharifi-Rad M., Contreras M.D.M., Segura-Carretero A., Fathi H., Nasrabadi N.N., Kobarfard F., Sharifi-Rad J. (2018). Thymol, thyme, and other plant sources: Health and potential uses. Phytother. Res..

[20] Khan S.T., Khan M., Ahmad J., Wahab R., Abd-Elkader O.H., Musarrat J., Alkhathlan H.Z., Al-Kedhairy A.A. (2017). Thymol and carvacrol induce autolysis, stress, growth inhibition and reduce the biofilm formation by *Streptococcus mutans*. AMB Express.

[21] Silva M.D., Sillankorva S. (2019). Otitis media pathogens—A life entrapped in biofilm communities. Crit. Rev. Microbiol..

[22] Idicula W.K., Jurcisek J.A., Cass N.D., Ali S., Goodman S.D., Elmaraghy C.A., Jatana K.R., Bakaletz L.O. (2016). Identification of biofilms in post-tympanostomy tube otorrhea. Laryngoscope.

[23] Donlan R.M., Costerton J.W. (2002). Biofilms: Survival mechanisms of clinically relevant microorganisms. Clin. Microbiol. Rev..

[24] Dickschat J.S. (2010). Quorum sensing and bacterial biofilms. Nat. Prod. Rep..

[25] Marino M., Bersani C., Comi G. (1999). Antimicrobial activity of the essential oils of *Thymus vulgaris* L. measured using a bioimpedometric method. J. Food Prot..

[26] Trombetta D., Castelli F., Sarpietro M.G., Venuti V., Cristani M., Daniele C., Saija A., Mazzanti G., Bisignano G. (2005). Mechanisms of antibacterial action of three monoterpenes. Antimicrob. Agents Chemother..

[27] Saptami K., Arokia Balaya Rex D., Chandrasekaran J., Rekha P.D. (2022). Competitive interaction of thymol with cviR inhibits quorum sensing and associated biofilm formation in *Chromobacterium violaceum*. Int. Microbiol..

[28] Miranda-Cadena K., Marcos-Arias C., Mateo E., Aguirre-Urizar J.M., Quindós G., Eraso E. (2021). In vitro activities of carvacrol, cinnamaldehyde and thymol against Candida biofilms. Biomed. Pharmacother..

[29] Walczak M., Michalska-Sionkowska M., Olkiewicz D., Tarnawska P., Warżyńska O. (2021). Potential of carvacrol and thymol in reducing biofilm formation on technical surfaces. Molecules.

[30] Valliammai A., Selvaraj A., Yuvashree U., Aravindraja C., Karutha Pandian S. (2020). sarA-dependent antibiofilm activity of thymol enhances the antibacterial efficacy of rifampicin against *Staphylococcus aureus*. Front. Microbiol..

[31] Martínez A., Manrique-Moreno M., Klaiss-Luna M.C., Stashenko E., Zafra G., Ortiz C. (2021). Effect of essential oils on growth inhibition, biofilm formation and membrane integrity of *Escherichia coli* and *Staphylococcus aureus*. Antibiotics.

[32] Cendra M.D.M., Torrents E. (2021). *Pseudomonas aeruginosa* biofilms and their partners in crime. Biotechnol Adv..

[33] Rosenfeld R.M., Schwartz S.R., Pynnonen M.A., Tunkel D.E., Hussey H.M., Fichera J.S., Grimes A.M., Hackell J.M., Harrison M.F., Haskell H. (2013). Clinical practice guideline: Tympanostomy tubes in children. Otolaryngol. Head Neck Surg..

[34] Meeran M.F., Jagadeesh G.S., Selvaraj P. (2016). Synthetic catecholamine triggers β1-adrenergic receptor activation and stimulates cardiotoxicity via oxidative stress mediated apoptotic cell death in rats: Abrogating action of thymol. Chem. Biol. Interact..

[35] Nagoor Meeran M.F., Javed H., Al Taee H., Azimullah S., Ojha S.K. (2017). Pharmacological properties and molecular mechanisms of Thymol: Prospects for its therapeutic potential and pharmaceutical development. Front. Pharmacol..

